# Effects of Endolithic Parasitism on Invasive and Indigenous Mussels in a Variable Physical Environment

**DOI:** 10.1371/journal.pone.0006560

**Published:** 2009-08-10

**Authors:** Gerardo Ivan Zardi, Katy Rebecca Nicastro, Christopher David McQuaid, Marcos Gektidis

**Affiliations:** 1 Department of Zoology & Entomology, Rhodes University, Grahamstown, South Africa; 2 Institute of Paleontology, Friedrich Alexander University, Erlangen, Germany; Smithsonian Institution, National Museum of Natural History, United States of America

## Abstract

Biotic stress may operate in concert with physical environmental conditions to limit or facilitate invasion processes while altering competitive interactions between invaders and native species. Here, we examine how endolithic parasitism of an invasive and an indigenous mussel species acts in synergy with abiotic conditions of the habitat. Our results show that the invasive *Mytilus galloprovincialis* is more infested than the native *Perna perna* and this difference is probably due to the greater thickness of the protective outer-layer of the shell of the indigenous species. Higher abrasion due to waves on the open coast could account for dissimilarities in degree of infestation between bays and the more wave-exposed open coast. Also micro-scale variations of light affected the level of endolithic parasitism, which was more intense at non-shaded sites. The higher levels of endolithic parasitism in *Mytilus* mirrored greater mortality rates attributed to parasitism in this species. Condition index, attachment strength and shell strength of both species were negatively affected by the parasites suggesting an energy trade-off between the need to repair the damaged shell and the other physiological parameters. We suggest that, because it has a lower attachment strength and a thinner shell, the invasiveness of *M. galloprovincialis* will be limited at sun and wave exposed locations where endolithic activity, shell scouring and risk of dislodgement are high. These results underline the crucial role of physical environment in regulating biotic stress, and how these physical-biological interactions may explain site-to-site variability of competitive balances between invasive and indigenous species.

## Introduction

Biological invasions are affecting the earth's ecosystems profoundly [1], and a major challenge in invasion biology lies in identifying the processes regulating invasion success [2], [3], [4], [5]. After the establishment of an invader, the demographic expansion of the species is independently or interactively affected by multiple biotic factors of the recipient environment [e. g. 6], [7], [8], [9]. Biotic interactions are widely modified by abiotic conditions [10], [11], [12]. Therefore, spatial and temporal variation of these conditions can modulate the strength of biotic interactions and indirectly influence the pattern of invasion. In this study, we demonstrate the importance of endolithic parasitation acting in concert with the physical habitat in limiting the invasive success of the mussel *Mytilus galloprovincialis* and in setting patterns of co-existence with the native mussel *Perna perna*.

Parasites play a crucial role in the hosts' ecology in plant and animal communities [13], [14]. They can have a direct, negative impact on host population density and growth rate [15], [16] or can indirectly interact with competition and predation [e. g. 17], [18]. The role of parasites can be critical in biological invasions because they can affect the proliferation of an invader and its interaction with native competitors, shifting competitive dominance from one species to another [19], [20]. Parasites in the recipient range can affect the establishment and spread of an introduced species either directly, or operating in concert with environmental conditions to determine the local success of an invader [21], [22].

The Mediterranean mussel *M. galloprovincialis* is a successful invader worldwide, and it is the most successful marine invasive species in South Africa [23]. On the south coast of South Africa, it co-exists with the indigenous *P. perna* on the low shore (referred to here as the mussel zone) in the intertidal. The upper and the lower areas of the mussel zone are dominated by *M. galloprovincialis* and *P. perna* respectively, while the two species overlap in the mid-mussel zone [24].

Microbial endoliths participate in many geologically and ecologically significant processes, including the production of fine grain sediment, bioerosion of limestone and other calcareous substrates such as shells, and the skeletons of living or dead animals and plants [25], [26], [27], [28]. Through their activity, they are able to inflict damage on their hosts [16], [29], [30].

In mussels, photoautotrophic endolithic infestation can be responsible for 50% of total mortality. The key pressure exerted by endolithic parasitation may, however, not result from catastrophic outbreaks but from sub-lethal effects, which, in an invasive scenario, can affect native-invader interactions and community structure. In their native range, infested mussels can have reduced condition index, shell growth and reduce shell strength [16], [31]. To repair the damage caused by endoliths, the host must increase the rate of shell thickening. Shell repair is an energetically demanding process that can alter the energetic budgeting of an organism and can reduce the energy available for reproduction and growth [32], [33].

The ecological outcomes of interactions between two species, such as mutualism and parasitism, often vary spatially and depend on the abiotic context in which those interactions occur [34], [35]. In mussels, the incidence of infested shells varies significantly over different spatial scales, being greater at higher tidal levels than on the low shore [36]. The majority of shells on the low shore have an intact periostracum, while at higher tidal heights the outer shell layer is often heavily abraded. Several studies indicate that the removal of the periostracum is a prerequisite to endolithic infestation [36], [37], [38]. Greater damage of this layer on the high shore may be related to higher hydrodynamic and wind stress experienced on the high shore [36].

The limiting factor for the distribution of photoautotrophic endoliths is light and this is reflected in the reduction of species diversity with increasing depth of water [39], [40]. Autotrophic endoliths prefer supra- and intertidal habitats [41], while shallow waters are inhabited by all known endolithic taxa in more or less equal proportions [40], [42]. However, due to the three-dimensional structure of the intertidal framework, light is not a constant factor. Shading has a large impact on the intertidal endolithic community, which, at shaded sites, is characterised by the absence of approximately 60% of the cyanobacteria species and its composition is comparable to an upper shallow-water community [43].

We hypothesise that endolithic infestation can be modulated by varying environmental conditions over large and small spatial scales and that differences in the degree of endolithic parasitation of competing invasive and indigenous species can shift competitive dominance from one species to another. In particular, we explicitly tested the following hypotheses: (1) the impact and incidence of infestation on *M. galloprovincialis* and *P. perna* populations will be higher on the open coast than within bays because of higher hydrodynamic stress; (2) at smaller scales (meters), the incidence of infested shells will be lower in shaded areas, because low light exposure will limit the development of photoautotrophic endoliths; (3) endolithic infestation will weaken shell strength and the re-routing of energy due to shell repair negative affects mussels condition index and attachment strength; (4) the species with higher degree of parasitism will suffer greater lethal and sub-lethal effects.

## Materials and Methods

### Incidence of endoliths

#### Meso-scale comparison between bay and open coast

**Figure 1:**
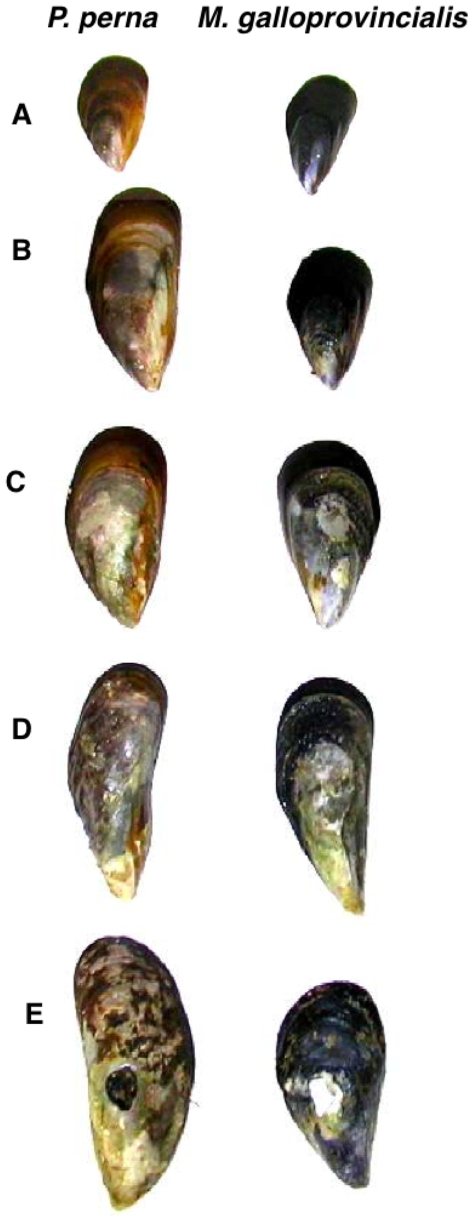
Examples of shells at varying stages of endolith infestation. Group A, shells with clean, intact periostracum and distinct outer lines; Group B, shells with central portion of surface eroding, outer striation on periostracum becoming indistinct; Group C, shells with erosion spreading past central portion, grooves and pits appearing on the shell surface; Group D, shells heavily pitted and becoming deformed, outer striation on periostracum almost completely absent; Group E, shells extremely pitted, deformed and brittle, eventually holed.

The incidence of endoliths was measured in two bays, Plettenberg Bay and Algoa Bay (34°00′17″ S, 23°27′17″ E, 33°58′47″ S, 25°39′30″ E), which are approximately 220 km apart on the south coast of South Africa, and at two open coast locations, Robberg and Cape Recife (34°06′14″ S, 23°23′07″ E, 34°02′27″ S, 25°32′01″ E), approximately 10 km to the west of Plettenberg Bay and Algoa Bay respectively. Each location included two sites 200 m apart at which three quadrats (15×15 cm) were haphazardly placed in the mid-mussel zone in sun-exposed areas (see below for definition) with 100% mussel cover. All mussels inside each quadrat were brought to the laboratory and separated by species. Mussels were individually measured (shell length) and separated into 10 mm size classes. Infestation severity was assessed subjectively by placing them into five categories, depending on degree of infestation: Group A, shells with clean, intact periostracum and distinct outer lines; Group B, shells with central portion of surface eroding, outer striations on periostracum becoming indistinct; Group C, shells with erosion spreading past central portion, grooves and pits appearing on the shell surface; Group D, shells heavily pitted and becoming deformed, outer striations on periostracum almost completely absent; Group E, shells extremely pitted, deformed and brittle, eventually holed (Fig. 1). The proportion of infested shells (i. e. all the mussels from group B to group E) fulfilled the requirements for parametric analysis (Cochran's Test) and was analysed using nested ANOVA (GMAV5 software) with species and habitat (bay or open coast) as fixed factors, and location nested within habitat, site nested within location (all random factors).

#### Micro-scale comparison between shaded and non-shaded sites

The incidence of endoliths was measured at Robberg at two sites 200 m apart. At each site, six quadrats (15×15 cm) were haphazardly placed in the mid-mussel zone in areas with 100% mussel cover in shaded and non-shaded areas (three quadrats each area) with the same wave exposure (i. e. same tidal height, lack of protecting structures in front of the area, same orientation towards the incoming waves, same shoreline angle) to avoid different scouring effects of waves between the two types of areas. Shaded areas were classified as cool sites that were estimated to be exposed to solar radiation for<25% of the day and, during high sun intensity (i. e. spring sunny day, 10a.m. to 4p.m.), experiencing PAR of 0–0.3 µmol m^−2^ s^−1^. Non-shaded or sun-exposed areas were classified as surfaces with limited shading that are likely to be exposed to solar radiation>60% of the day and, during high sun intensity (as above), experiencing PAR higher than 0.13 µmol m^−2^ s^−1^. The PAR values were estimated with an Integrating Quantum/Radiometer/Photometer (Li-188B). Mussels inside each quadrat were brought to the laboratory, separated by species and processed as above. The proportion of infested shells (groups B to E) was analysed with a 2-way ANOVA with shade (shaded and non-shaded) and species as fixed factors.

### Sub-lethal effects of endolithic infestation

Adult mussels of shell length 4–5 cm of both species belonging to Groups A and D were collected at Robberg and brought to the laboratory to be processed.

#### Shell strength

Clean (Group A) and infested (Group D) mussel shells (n = 50, for each group for each species) were tested for strength. The compressive force required to crack the shell was measured using a recording spring scale (Chatillon-N.Y.-U.S.A.-MODELIN-25 Clean (Group A) and infested (Group D) mussel shells (n = 50, for each group for each species) were tested for strength. One valve from each mussel shell was placed horizontally on a plane surface in which there was a narrow slot running at right angles to the valve length. A small (15×10×3 mm) metal plaque was set on the valve on the site of the posterior adductor muscle. The plaque had a small hole at either end from which a length of fishing line ran through the slot so that the lines passed on either side of the shell. The lines led to a mechanical, recording spring scale underneath the plane surface. The spring scale was steadily and uniformly pulled downward, normal to the plane surface. As the compressive force in Newtons required to break the shell was applied over a small area, it was taken only as a relative and not absolute estimate of shell strength. The shell strength was analysed with a 2-way ANOVA with endolith infestation (group A or D) and species as fixed factors.

#### Attachment strength

*Mytilus galloprovincialis* and *Perna perna* individuals (n = 15 for each species, each infestation group) were tested *in situ* for attachment strength. A 2 mm diameter hole was carefully drilled with a hand-held battery drill through the shell valves close to the posterior margin without damaging any byssal threads. A fishhook was inserted through the hole and connected to a recording spring scale (Chatillon-N.Y.-U.S.A.-MODELIN-25) via a fishing line. Mussels living within a mussel bed are mainly subjected to lift forces, which act perpendicularly to the substratum [44], thus the scale was lifted normal to the rock surface until dislodgment occurred (1–3 s) and the force required to detach each mussel was recorded in Newtons (N). All dislodged mussels were at least 20 cm from each other so that attachment strength measurements were not influenced by previous ones. Data were analysed using a 2-way ANOVA with the presence of endoliths (group A or D) and species as a fixed factors.

#### Condition index

The wet body was dissected from the valves (n = 50 for each species, each group) and dried at 60°C to a constant weight. Samples were weighed to the nearest 0.001 g and the condition index (CI) was calculated using the following equation from Davenport and Chen [45]:
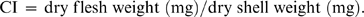



This measurement was performed in July (2007) and repeated in September (2007). The data were analysed with a 3-way ANOVA with endolith infestation (group A or D), species and month (July and September) as fixed factors.

### Lethal effects of endolithic infestation

Mussel mortality was investigated by clearing five 1 m×1 m quadrats of all dead (gaping widely during low tide) mussels. Quadrats were placed haphazardly at Robberg over 100 m of coast in sun-exposed areas with a 100% mussel cover in the mid-mussel zone. In order to ensure that only recent mortalities were included in the survey, only shells with a shiny inner nacreous layer were used. The insides of mussel shells become heavily fouled within a month after death and all such shells were discarded. Endoliths cause a distinctive discoloration, dissolution and finally fracturing of the shell around the site of adductor muscle attachment [36]. All shells exhibiting these fracture holes were assumed to have died from endolithic activity. Data were analysed using a 1-way ANOVA with species as fixed factor and percentage mussel mortality attributed to endolithic activity as the dependent factor.

### Shell thickness

Clean shells (i. e. Group A; n = 20 each species) were embedded in resin and sectioned longitudinally along the anterior-posterior axis using a diamond saw. An industrial rotating sander with different grades of sanding discs was used to smooth the cut surface before polishing to a blemish-free surface with household metal polish. Shells were observed under a dissecting microscope (50×) and the widths of the whole shell and of the periostracum above the adductor muscle were measured to the nearest 0.01 mm using an ocular eyepiece. The data were analysed using 1-way ANOVA with species as a fixed factor for the shell width and for the periostracum separately.

## Results

### Incidence of endoliths

#### Meso-scale comparison between bays and open coast

**Figure 2:**
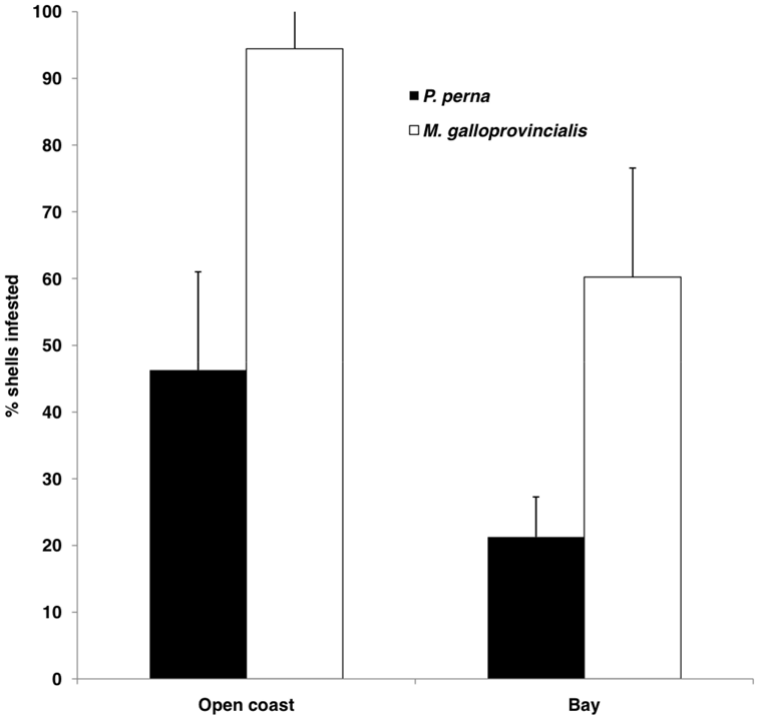
Incidence of endoliths: meso-scale comparison between bays and open coast.

**Figure 3:**
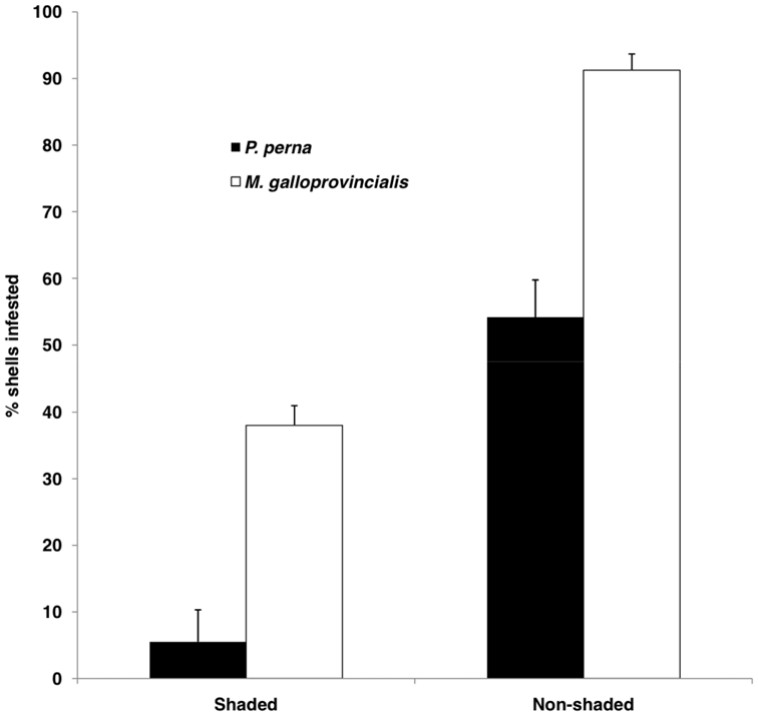
Degrees of infestation of different size classes: micro-scale comparison between bays and open coast. Proportions of shells exhibiting different degrees of infestation severity (Groups A–E, see Fig. 1) and grouped into 10 mm size classes. Infestation severity was determined in the mid-mussel zone at (a) bay and (b) open coast habitats for *P. Perna* and at (c) bay and (d) open coast for *M. galloprovincialis.*

*Mytilus galloprovincialis* always had a significantly (p<0.05; Fig. 2) higher proportion of infested shells than *Perna perna*, while significantly (p<0.05) more mussels on the open coast had endoliths than in bay habitats. *M. galloprovincialis* mussels were 48.5% and 38.2% more infested than *P. perna*, on the open coast and in bay habitats respectively, while infestation increased with shell length (Fig. 3).

Initial infestation (Group B, see Fig. 1) of *P. perna* occurred in mussels 10–20 and 20–30 mm in length, at open coast and bay habitats respectively. By the time they had reached 50 to 60 mm in length, 100% of *P. perna* on the open coast exhibited endolith-induced erosion and most of the shells were heavily or extremely pitted (Groups C and D). In bays, about 25% of the 50–60 mm shells were not infested, while the other shells exhibited Group C and D deformation. None of the *P. perna* shells examined in this experiment were holed because of endolithic infestation.

Initial infestation of the invasive species was visible in shells smaller than 1 cm. Most of the *M. galloprovincialis* 40–50 mm in shell length had Group C or D deformation (Fig. 3c,d). On the open coast, endolith-induced shell fractures (Group E) occurred in mussel size class 30 to 40 mm and above; once grown to this size, more than 20% exhibited holes related to endolithic infestation.

#### Micro-scale comparison between shaded and non-shaded sites

**Figure 4:**
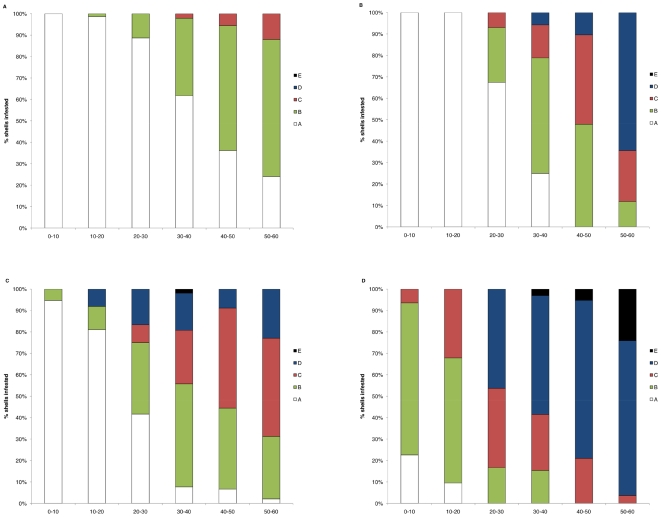
Incidence of endoliths: micro-scale comparison between shaded and non-shaded sites. Mean (+SD) percentage of *M. galloprovincialis* and *P. perna* shells that exhibited damage induced by endolithic infestation, at shaded and non-shaded sites.

At both shaded and non-shaded sites, *M. galloprovincialis* had a significantly higher proportion of infested shells than *P. perna* collected at the same sites (p<0.001) *M. galloprovincialis* mussels were 32.5% and 37% more infested than *P. perna*, at shaded and non-shaded sites respectively (Fig. 4).

**Figure 5:**
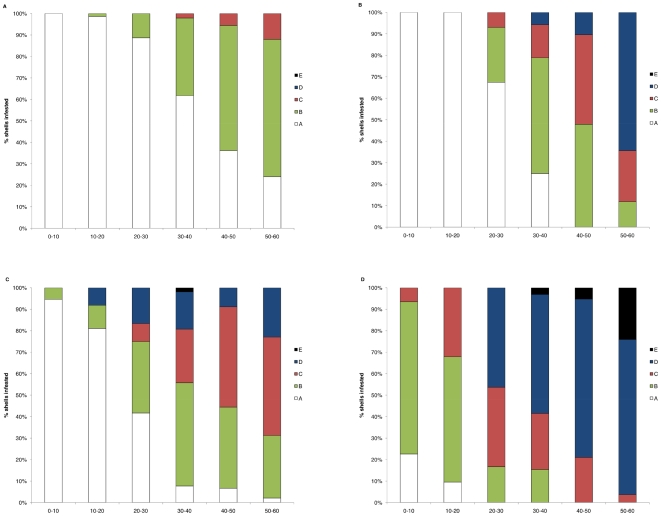
Degrees of infestation of different size classes: micro-scale comparison between shaded and non-shaded sites. Proportions of shells exhibiting different degrees of infestation severity (Groups A–E, see Fig. 1) and grouped into 10 mm size classes. Infestation severity was determined in the mid-mussel zone at (a) shaded and (b) non-shaded sites for *P. Perna* and at (c) shaded and (d) non-shaded sites for *M. galloprovincialis*.

Infestation increased with shell length and was more severe at non-shaded than at shaded sites for both species (p<0.001; Fig. 5).

Initial infestation (Group B) of *P. perna* occurred in mussels 10–20 and 30–40 mm in length, at non-shaded and shaded sites respectively. By the time they had reached 40–50 mm in length, 100% of *P. perna* at non-shaded sites exhibited endolith-induced erosion and at 50–60 mm more than 50% of the shells were heavily or extremely pitted (Group D and E). At shaded sites, more than 60% of the 50–60 mm in length shells was not infested, while the other shells exhibited only Group B deformation.

In the invasive species, Group B and C deformation was visible in shells smaller than 10 mm at non-shaded sites, while, when shaded, initial infestation (Group B) occurred only in mussels longer than 10 mm. No non-infested mussels (i.e. Group A) were recorded for sizes bigger than 40 and 20 mm at shaded and non-shaded sites respectively. At non-shaded sites, endolith-induced shell fractures (Group E) occurred in size classes 30–40 mm and above; once grown to the maximum size, more than 50% exhibited holes related to endolithic infestation.

### Sub-lethal effects of endolithic infestation

#### Shell strength

**Figure 6:**
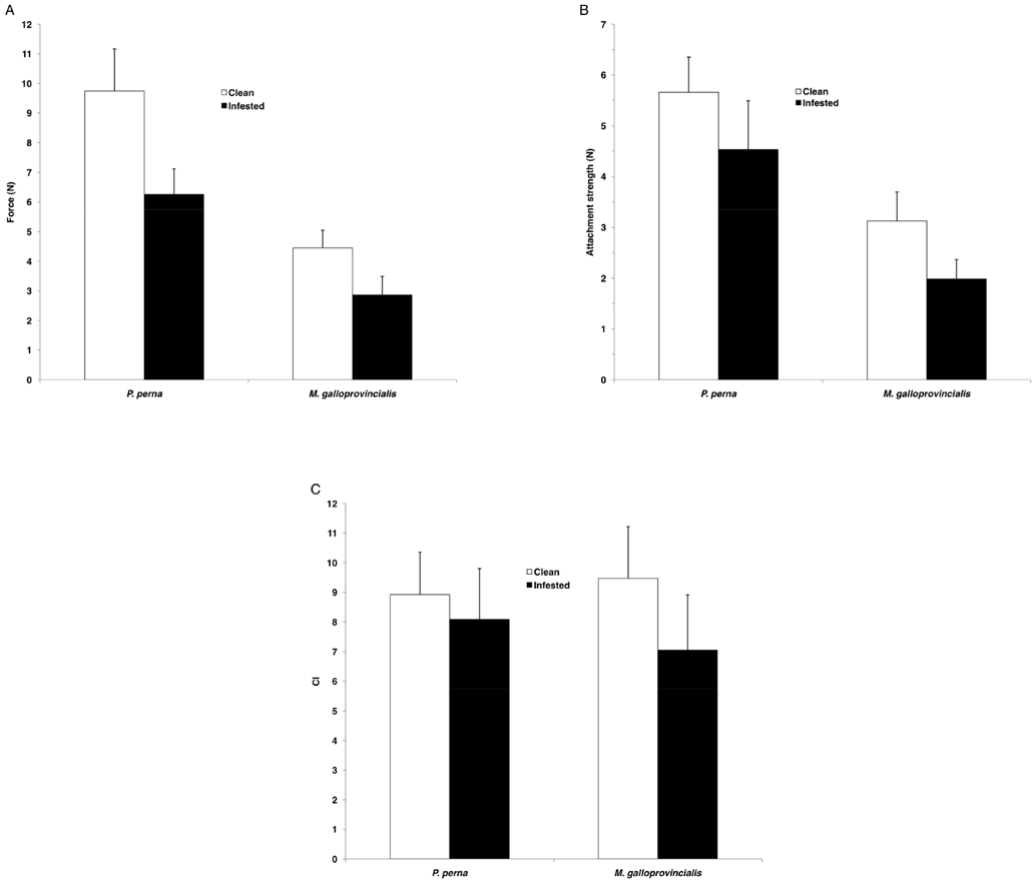
Sub-lethal effects of endolithic infestation. Sub-lethal effects of endolithic infestation in *P. perna* and *M. galloprovincialis* infested (Group D) and clean (Group A) mussels: (a) mean (+SD) force required to break shells; (b) mean (+SD) attachment strength; (c) mean (+SD) condition index, months pooled.

Within each group, *P. perna* shell strength was significantly higher than that of *M. galloprovincialis* (square root transformation; SNK test, p<0.001; Fig. 6a). Within each species, there was a significant effect of endoliths (p<0.001), with the shell being more robust when not infested. Endolithic colonization reduced shell strength by a mean of 35% in both species.

#### Attachment strength

When infested, both species had lower attachment strength than clean mussels (p<0.001; Fig. 6b), and the differences in attachment strength between the two species was maintained (*P. perna*>*M. galloprovincialis*, p<0.001). Endolithic colonization reduced attachment strength by a mean of 19.9% in *P. perna* and 36.5% in *M. galloprovincialis*.

#### Condition index

Both species showed significant effects of endolithic infestation on condition in July and September (p<0.001; Fig. 6c). The difference between CI of infested (Group D) and clean (Group A) mussels was on average 23% for *M. galloprovincialis* and for 9.4% *P. perna*.

### Lethal effects of endolithic infestation

**Figure 7:**
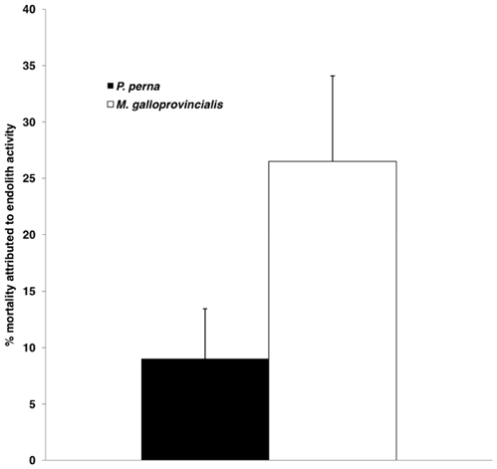
Lethal effects of endolithic infestation. Mean percentage of total mortality (+SD) attributed to endolith-induced fracture holes in *P. perna* and *M. galloprovincialis*.

*M. galloprovincialis* mortality rates were significantly higher (p<0.01) than *P. perna* values. A total of 190 recently dead mussels were collected from five 1 m×1 m quadrats. Of these shells, 26.5% and 9% exhibited large endolith-induced, probably lethal fracture holes for *M. galloprovincialis* and *P. perna* respectively (Fig. 7).

### Shell thickness

**Figure 8:**
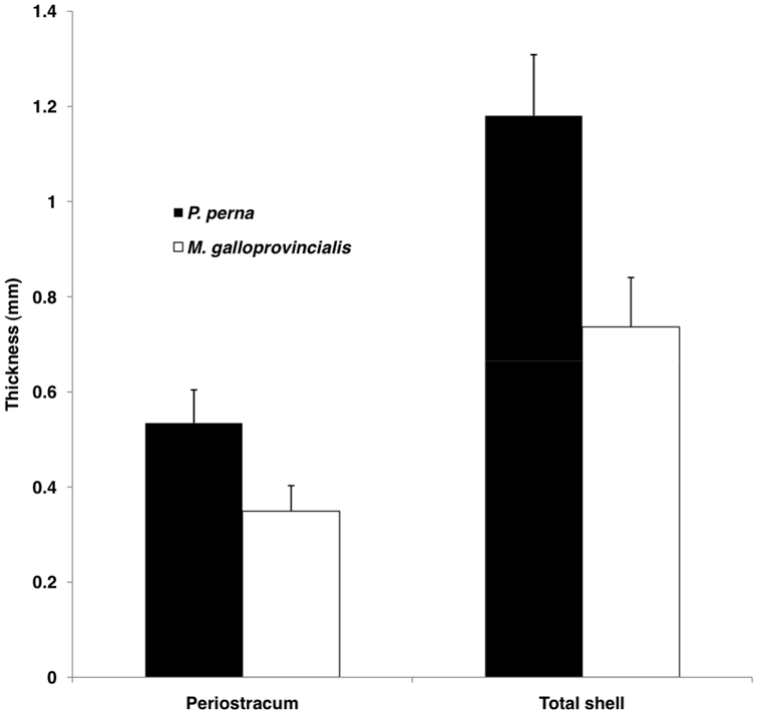
Shell thickness. Mean (+SD) periostracum and total thickeness of shells of *M. galloprovincialis* and *P. perna*.

Both periostracum and whole shell were significantly thicker for *P. perna* than for *M. galloprovincialis* (p<0.001 in both cases; Fig. 8). The periostracum and shell of *M. galloprovincialis* were approximately 35% thinner than in *P. perna*.

## Discussion

Our results demonstrate a synergistic effect of biotic stress and physical environment on the coexistence between an invasive and a native species. For invasive species, parasites can be considered as ecological filters through which invaders must pass. These same filters will also act on native competitors, and parasites which have different effects on native and introduced species can shift competitive dominance from one species to another [14], [19]. For example, in South Africa, two trematode species castrate and reduce the growth of *Perna perna* consequently limiting its competitive abilities against *Mytilus galloprovincialis*, which is not infected [17], [46]. In the same way, introduced live-bearing fishes in Australia had a much lower number of parasite species than ecologically similar native fish, which may give them a competitive advantage [47]. Here we show that endolith infestation of the shell occurs in both the invasive and in the indigenous species, but with higher incidences in *M. galloprovincialis*. Through competition experiments conducted in the field, earlier studies have shown that *P. perna* initially facilitates survival of the invasive *M. galloprovincialis* in the low mussel zone by providing protection against waves, but later excludes *M. galloprovincialis* through interference competition for space [48]. In the mid zone *M. galloprovincialis* weakly facilitates *P. perna* [49]. In this context, areas with high infestation levels could even more strongly favour the native species in the low mussel zone and perhaps create new balances in the mid zone.

The initial colonization of shells also varied between the two species, with endolith infestation never occurring in the smallest size classes of *P. perna*, while *M. galloprovincialis* as small as recruits were attacked by endoliths. In larger individuals, endoliths spread throughout the shell, causing progressive damage and, particularly in *M. galloprovincialis*, resulted in localised shell disintegration. The two species have different growth rates [50], and therefore there could be some confounding effects when comparing species within the same size class as they may not be the same age. Nevertheless, when pooling all size classes the inter-species differences are maintained. The activity of photoendoliths can result in the mortality of a large number of adult mussels [16], [38]. The higher prevalence of parasites in one host than in a sympatric host can explain the differences in lethal rates between these two species. We show that endolithic activity contributed to mortality rates of both species but *M. galloprovincialis* mortality rates were significantly higher than those of *P. perna*. Given the high variability of endolithic infestation along the South African coastline, this result must be interpreted only in terms of a comparison between the two species.

The effects of parasitism also varied over small and large spatial scales. Both species exhibited higher incidences of infested shells (up to 70%) on the open coast than within bays (as low as 30%). Greater wave action on the open coast [51] compared to sites within bays may result in an increase in shell erosion both through contact between adjacent mussels and the abrasion of shells by sediment carried by more powerful waves. This increase in erosion may result in greater damage to the periostracum of shells, a prerequisite to endolithic infestation [36], [37], [38], thus strongly influencing the frequency of infestations along coastal wave exposure gradients.

Since many micro-borers are photosynthetic [39], a key factor in the development of species composition of endolithic assemblages is the degree of light exposure. Numerous studies have examined the bathymetric distribution of micro-borers [e. g. 40], [52], [53]. However, it is surprising that only a few studies have examined between-site variations in the composition of micro-endolithic assemblages [42], [43]. The depth range of micro-endolithic assemblages is reduced in turbid waters compared to clear-water sites [42]. When analysing micro-endolithic community patterns in experimental carbonate blocks at shaded and non-shaded intertidal habitats, a strong effect of shading is observed [43]. Here, we demonstrate that shaded sites have a diminished infestation frequency compared to non-shaded sites, indicating an important role for micro-scale variation of sun exposure in the control of endolithic infestation. Even though the three most abundant cyanobacteria species infesting *P. perna* shells on South African rocky shores can be found at depths of<40 m [36], their photosynthetic activity, and consequently their boring activity, are likely to be influenced by light and to be higher at non-shaded intertidal sites. Other biotic factors, such as differences in the abundance of macro-borers and grazers at shaded and sun-exposed sites, could also play a role [see 54]. For example, endolithic activity attracts grazing by gastropods and abrasion by grazing organisms in turn stimulates endolithic cyanobacterial erosion [26].

Shell strength of both species was weakened by the presence of endoliths, but the invasive *M. galloprovincialis* has a thinner, weaker shell than *P. perna*, enhancing the negative effects of endolithic boring. Shell is a defence against shell-crushing predators and the breaking action of waves [55], [56]. Mussels with weakened shells are more vulnerable to both predation and the mechanical effects of wave action [38]. This, together with our results, suggests that endoliths will have an indirect negative effect on mussels through an increased disturbance effect, and that this effect will be greater for the invasive species.

Condition index (CI), which relates the flesh weight to the amount of shell, is an important measure of the physiological status of mussels and the relative allocation of resources to tissue or shell growth [57], [58]. Previous studies showed that the flesh mass of infested mussels is significantly lower than that of non-infested individuals [16], [31]. This difference was almost entirely due to differences in gonad mass, which was approximately 100% higher for non-infested mussels [16]. Our results indicate that, endolitholitic infestation negatively affect CI of both *P. perna* and *M. galloprovincialis*, and that the negative effect is greater for the invasive species. This suggests that endoliths limit the development of reproductive tissue and that the need to invest heavily in reproduction is outweighed by the more urgent need to maintain the shell. Other studies have shown a trade off between physiological needs in these mussels. In winter and on the open coast, both species invest more energy in attachment strength, but *P. perna* can accommodate the energetic demands of increased byssal production without altering gonad production, while *M. galloprovincialis* cannot [59]. Here we show that the invader is debilitated not only by the more extreme wave action on the open coast but also by endolith activity. The more stressful and energetically demanding open coast habitat acts negatively on the physiological performance of *M. galloprovincialis*. One consequence of this is that, while both species are more abundant in bays than on the more wave exposed open coast, this effect is stronger for *M. galloprovincialis* than for the indigenous *P. perna* [60].

Mussels attach to the substratum by mean of a bunch of collagenous threads called the byssus [61] and the process of byssal thread production can be energetically expensive, forming 8 to 15% of a mussels' monthly energetic expenditure [62]. Shell deposition and repair are also very energy demanding; mussels invest up to 25 to 50% of the total body energy in this process [62], [63]. Infested mussels had significantly lower attachment strengths than clean mussels, suggesting that the need to repair the shell limits the energy available for the production of byssal threads. Moreover, lower attachment strength of infested mussels could increase the risk of dislodgment, especially on the open coast where wave action is greater [51].

Overall, it is likely that the negative consequences of a trade-off between an intact shell, strong attachment and a high CI will be particularly marked on the open coast, where shell scouring (leading to endolith attack) and the risk of dislodgment are both high and this will be particularly true for the invasive species. In fact, the Mediterranean mussel *M. galloprovincialis* is a very successful invader worldwide [23], but high hydrodynamic stress reduces its competitive abilities, increasing its mortality rates and reducing its reproductive output [59 , authors unpubl. data]. Here large-scale coastal topography and differences between habitats in sun exposure influence the degree of endolithic colonization and synergistically affect the physiology of the two species, probably creating new competitive balances which could be tested by competition experiments between infested and non-infested individuals. Nevertheless, we show here how our understanding of the dynamics of coexistence between invasive and indigenous species must take into account the complex interactions between environmental heterogeneity and biological processes. This approach will help to create general schemes of the seemingly idiosyncratic patterns of invasion.
